# Water printing of ferroelectric polarization

**DOI:** 10.1038/s41467-018-06369-w

**Published:** 2018-09-18

**Authors:** Yu Tian, Lanying Wei, Qinghua Zhang, Houbing Huang, Yuelin Zhang, Hua Zhou, Fengjie Ma, Lin Gu, Sheng Meng, Long-Qing Chen, Ce-Wen Nan, Jinxing Zhang

**Affiliations:** 1 Department of Physics, Beijing Normal University, Beijing, 100875 China; 2 Institute of Physics, Chinese Academy of Science, Beijing National Laboratory of Condensed Matter Physics, Beijing, 100190 China; 3 Advanced Research Institute of Multidisciplinary Science, Beijing Institute of Technology, Beijing, 100081 China; 4 Department of Materials Science and Engineering, The Pennsylvania State University, University Park, State College, PA 16802 USA; 5 State Key Lab of New Ceramics and Fine Processing, School of Materials Science and Engineering, Tsinghua University, Beijing, 100084 China

## Abstract

Ferroelectrics, which generate a switchable electric field across the solid–liquid interface, may provide a platform to control chemical reactions (physical properties) using physical fields (chemical stimuli). However, it is challenging to in-situ control such polarization-induced interfacial chemical structure and electric field. Here, we report that construction of chemical bonds at the surface of ferroelectric BiFeO_3_ in aqueous solution leads to a reversible bulk polarization switching. Combining piezoresponse (electrostatic) force microscopy, X-ray photoelectron spectroscopy, scanning transmission electron microscopy, first-principles calculations and phase-field simulations, we discover that the reversible polarization switching is ascribed to the sufficient formation of polarization-selective chemical bonds at its surface, which decreases the interfacial chemical energy. Therefore, the bulk electrostatic energy can be effectively tuned by H^+^/OH^−^ concentration. This water-induced ferroelectric switching allows us to construct large-scale type-printing of polarization using green energy and opens up new opportunities for sensing, high-efficient catalysis, and data storage.

## Abstract (web summary)

Controlling ferroelectric polarization is conventionally achieved by applying electric fields, mechanical force or similar. Here reversible switching of the bulk polarization of a BiFeO_3_ thin film is demonstrated by pattering aqueous solution on to the surface enabling large-scale switching.

## Introduction

Over the past century, exploration and artificial control of molecular/ion absorption, charge transfer and associated structures across the solid–liquid interface^1,2^ is of particular interest for diverse communities including life science^3^, chemical catalyzing^4^, efficient energy transition and storage^1,5–7^. Benefiting from the non-volatile/reversible electric polarization and high charge density at surfaces^8^, ferroelectrics could be a distinguished model system to artificially construct and switch the solid–liquid interfacial states in liquid environment^9–12^, which has been proposed to play critical roles on facilitating the energy storage^13^, cells proliferation^14^, and chemical reactions^15–17^ etc. However, it is usually challenging to in-situ control such ferroelectric–liquid interfacial structures, which severely inhibit the energy transformation with high efficiencies^15^. Ferroelectric polarization is usually switched using external physical methods (e.g., electric field, mechanical force, ionic implantation)^18–21^, which not only consumes external fossil energy, but also fails to in-situ design the desired ferroelectric–liquid interfacial structures. Therefore, understanding and control of the ionic and polar structures across the ferroelectric/liquid interface by in-situ switching the bulk polarization, vice versa, are fundamentally important so that a controllable/dynamic charge transfer in accompany with an efficient energy conversion across the interface may be achieved.

Although it has been recognized that the polarization of ferroelectrics may potentially determine their surface chemistry over 60 years ago^22^, the converse effect remains a mystery. Multiple physical and chemical processes, e.g., molecular adsorption^23–25^, the concomitant electron transfer^26^, were proposed to occur at the surface of ferroelectrics when it was exposed to liquids, which may change the surface polarity (e.g., LiNbO_3_, BaTiO_3_)^23,27^. However, the charge transfer process becomes very complicated because both molecular/ion adsorption and dissociative adsorption coexist on these polar surfaces^23^. Therefore, owing to the obscure insights of the chemical structure and energy conversion across the interface, reversible control of the bulk ferroelectric polarization by designing the interfacial chemical structures in liquids (especially water which is described as the ‘‘universal solvent’’ in chemistry and vital for all lives and nature^4^), and vice versa, remains a big challenge.

In ferroelectrics, bismuth ferrite (BiFeO_3_; BFO), with a large polarization^28,29^, was found to be a multiferroic materials^30,31^ with high stability^32^ and bio-compatibility^10^. A high-lying valence band composed of the Bi 6*s* and O 2*p* atomic orbitals results in a band gap within visible light (*E*_g_≈2.6−2.8 eV)^33–35^, and the energy bands straddle the water redox levels, suggesting its unique potential for water splitting^36^. Therefore, comparing to other ferroelectric oxides, water molecules are more likely to be dissociative adsorbed on its polar surface in order to construct controllable ionic/molecular structures and associated energy conversion across the interface.

In this paper, we report that a reversible large-area switching of bulk polarization occurs via artificial design of ion adsorption and chemical reaction across BFO–water interface, which is assisted by tuning H^+^/OH^−^ concentration in aqueous solution. We discover that a significant formation of controllable chemical bonds (metal-O-H at BFO surface) driven by large surface electric potential gives rise to a strong atomic displacement of Fe atom with respect to oxygen octahedral in its bulk, which has been evidenced by spectroscopic/microscopic measurements and understood by first-principles calculations. This deterministic conversion of the surface chemical energy and bulk electrostatic energy may provide a paradigm for designing controllable ferroelectric–liquid interfacial structures to efficiently tailor charge transfer or ionic adsorption process.

## Results

### Aqueous-solution-induced large-area switching of ferroelectric polarization

**Fig. 1:**
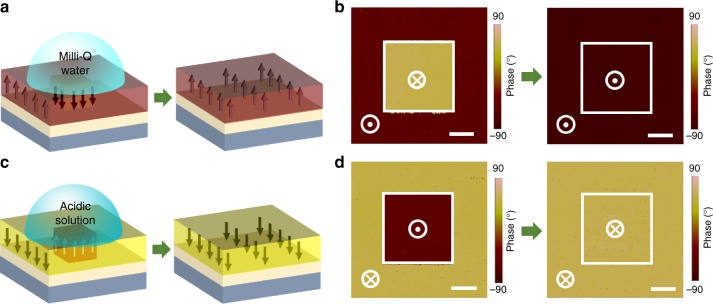
Aqueous-solution-induced reversal of ferroelectric polarization. Schematics of BiFeO_3_ (BFO) grown on a SrTiO_3_ (STO) substrate with a (La,Sr)MnO_3_ (LSMO) layer, switching from downward/upward to upward/downward after exposing the films to Milli-Q water (**a**) and acidic solution (**c**). The arrows in the schematics represent the out-of-plane component of the polarization. The initial bipolar structure in the left panel of **a** and **c** was generated by scanning the surface with a tip under +5 V and -5 V, respectively. **b**, **d** The corresponding piezoresponse force microscopy (PFM) images showing the polarization switching from downward/upward to upward/downward. Scale bar, 2 μm (**b**, **d**)

BFO thin films with switchable ferroelectric polarization were prepared on SrTiO_3_ (STO) substrates with conductive (La,Sr)MnO_3_ (LSMO) bottom layers. The initial out-of-plane projections of the ferroelectric polarization (upward or downward) can be artificially controlled by designing the termination of the conductive buffer layers^37,38^, which were characterized using piezoresponse force microscopy (PFM). As shown in Fig. 1a, when an initial upward-polarized BFO film with a downward ferroelectric domain (5 × 5 µm^2^) was exposed to a drop of water solution (Milli-Q water, pH = 7), the downward domain reversed to its initial upward state spontaneously, as characterized by PFM images in Fig. 1b. The whole switching process in water can be monitored in-situ by liquid PFM^39^, as shown in Supplementary Fig. 1. The same results were also observed when the sample was exposed to alkaline solution (pH > 7) as seen in Supplementary Fig. 2. On the other hand, when an initial downward-polarized BFO film with upward ferroelectric domain (5 × 5 µm^2^) was exposed to a drop of aqueous solution (Fig. 1c), the upward domain cannot reverse to its initial downward state until the aqueous solution was tuned to acidic aqueous solution (pH ~ 3), as demonstrated in the PFM images of Fig. 1d. For all above aqueous solution treatments, no degradation occurs, which can be characterized by high-quality atomic force microscopy (AFM) in Supplementary Fig. 3. The epitaxial growth recipes, PFM, liquid PFM and AFM measurements are included in Methods.

**Fig. 2:**
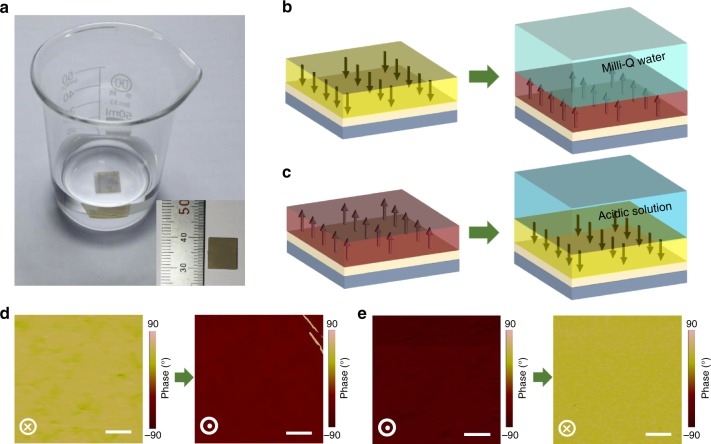
Aqueous-solution-induced reversal of whole film polarization. **a** Photograph of water treatment process and the corresponding sample size is 10 × 10 mm^2^ (inset). The polarizations of the whole films reversed to the upward (**b**) and downward (**c**) after exposing the film to Milli-Q water and acidic solution, respectively. The corresponding PFM images showing the polarization switching from downward/upward to upward/downward after Milli-Q water (**d**) and acidic-solution (**e**) treatments. Scale bar, 2 μm (**d**, **e**)

Interestingly, such an aqueous solution-induced polarization switching does not just occur locally. The overall polarization in the sample (10 × 10 mm^2^) reversed back to upward (downward) by water (acidic aqueous solution with pH = 3) treatment as illustrated in Fig. 2. And the polarization reversal was evinced by PFM at many regions as shown in Supplementary Fig. 4. Moreover, in contrary to the Milli-Q water treatment, no polarization reversal was observed when BFO films was exposed to other polar liquids with bare free H^+^ and OH^−^ ions such as acetone as seen in Supplementary Fig. 2. This implies that only physical adsorption of polar molecules in liquids on BFO surface may not induce the polarization reversal. These results give us a hint that the significant concentrations of active H^+^/OH^−^ ions in aqueous solution and ionic interaction across this solid–liquid interface may play critical roles on the polarization reversal.

### Surface structure of BFO after aqueous-solution treatments

**Fig. 3:**
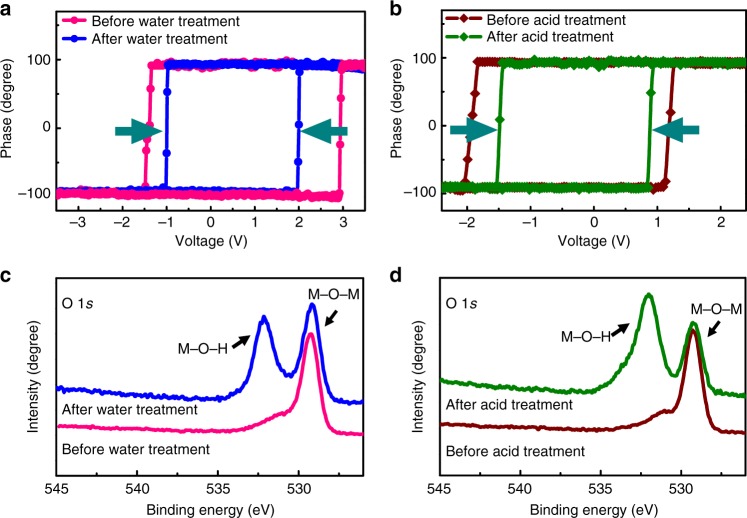
New chemical bond formation after aqueous-solution treatments. Corresponding typical quasi-static piezoresponse hysteresis loops show that coercive fields decreased after Milli-Q-water-induced polarization switching (**a**) and acidic-solution-induced polarization switching (**b**). X-ray photoelectron spectroscopy spectrums showing an appearance of peak belongs to M–O–H bond after the polarization reversed in Milli-Q water (**c**) and acidic solution (**d**), indicating a significant formation of new surface chemical structure on BFO

A straightforward way to study ferroelectric properties of the BFO thin films is the characterization of ferroelectric hysteresis behaviors before and after the aqueous-solution-induced polarization switching. Via local quasi-static hysteresis studies (Fig. 3a, b), a dramatic decrease of the coercive field (collected from average values from Supplementary Fig. 5) was discovered after the aqueous-solution-induced polarization switching, suggesting the formation of a new surface state on BFO. X-ray photoemission spectroscopy (XPS) is an effective technique to analyze the surface chemical construction with a few atomic layers^27^. Therefore, BFO thin films was further characterized using XPS before and after aqueous-solution-induced switching in order to reveal their surface structures (details can be seen in Methods). As shown in Fig. 3c, d, there appears dramatic enhancement of the photoemission peak (∼531.4 eV) in the spectra after the polarization was switched spontaneously in aqueous environments. In perovskite oxide materials, the peak with a binding energy of ∼529.1 eV corresponds to the lattice oxygen in perovskite BFO (metal-oxide-metal, M–O–M), and the emerging peak at higher binding energies of ∼531.4 eV indicates the presence of terminal hydroxyls (metal-oxide-hydrogen, M–O–H) with a high concentration^40,41^. The dramatic enhancement of surface M–O–H concentration when ferroelectric switching occurs in contact with water/acidic solutions indicates that there is a strong interplay between the surface electric potential and ionic interaction. Consequently, the ionic chemical reaction across the BFO/aqueous solution interface may be responsible for the polarization switching so that a new metastable state was stabilized by the formation of the new surface chemical bonds. These surface chemical bonds (new surface states) provide prevailing extrinsic charges that will decrease the coercive field, as proposed by previous theoretical work^42,43^, which is consistent with our local quasi-static hysteresis measurements.

### Mechanism of aqueous-solution-induced polarization switching

**Fig. 4:**
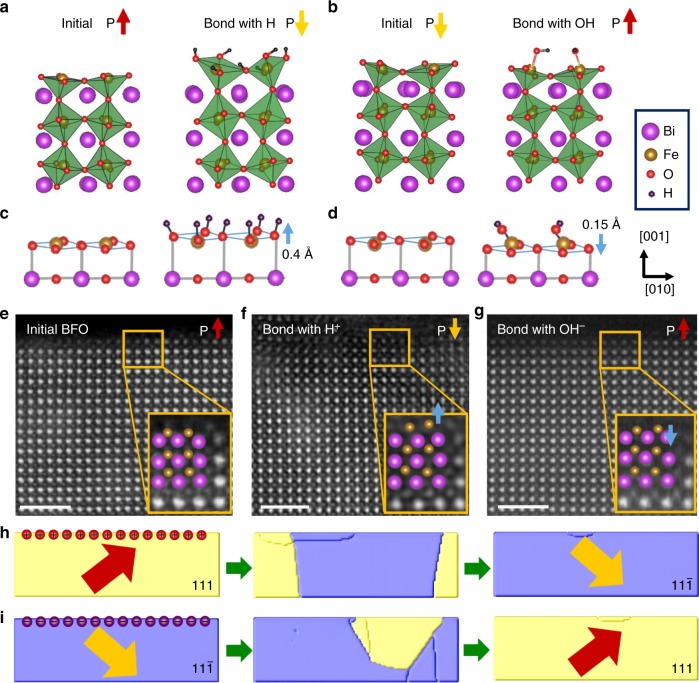
Chemical-bonding-induced switching of ferroelectric polarization. BFO polarizations are switched from upward/downward to downward/upward with adsorbed of four H (**a**) and two OH (**b**), respectively. BFO surface Fe and O atomic displacements along *z*-axis with four H (**c**) and two OH (**d**) adsorption by density function theory. Scanning transmission electron microscopy images of initial BFO thin film (**e**) after contact with acidic-solution (**f**) and Milli-Q water (**g**). The arrows in **a**, **b**, and **e** represent the out-of-plane component of the polarization. Scale bar, 2 nm (**e**). **h**, **i** Phase-field simulations of bulk polarization switching processes based on the emerging surface charge

To investigate physical/chemical essence of the interplay between surface electric potential and ionic concentrations, and the consequent polarization reversal at atomic level, density function theory (DFT) calculation (employing VASP package with PAW pseudopotentials and PBE functionals) and high-resolution scanning transmission electron microscopy (STEM) measurement were performed (see Methods). Ionic/molecular OH, H, H_2_O etc. are the major components in aqueous solution. The chemical adsorption energy can be expressed as:

1 $$\Delta H = E_{\mathrm{f}} + n\cdot E_{{\mathrm{ads}}} - E_{{\mathrm{coup}}}$$

where *E*_f_ is the total energy of the optimized n-layer bare BFO slab without water, *E*_ads_ is the total energy of the adsorbed atom or molecule, and *E*_coup_ is the total energy of the optimized n-layer slab with adsorbed species. Calculations demonstrate a larger released chemical adsorption energy with OH/H (>1.5 eV) adsorption than the one of H_2_O (~ 0.5 eV) molecules adsorption, which indicates that H/OH are more energetically favorable adsorbed on BFO surface comparing with H_2_O. Based on our electrostatic force microscopy (EFM) measurements (as shown in Methods), where there is a negative (positive) surface charge in upward-polarized (downward-polarized) BFO films as seen in Supplementary Fig. 6a, b. H^+^ and OH^−^ were preferred to be adsorbed by BFO with upward and downward polarizations respectively, as seen in Fig. 4a, b. Our first-principles calculations show that ionic reaction occurs to construct M–O–H bonds when H and OH are in contact with O and Fe in the energy-favorable FeO_2_ plane of BFO surface^44^, leading to the upward and downward atomic displacement of Fe ions with respect to their original states as seen in Fig. 4c, d. Such surface atomic displacements were further corroborated by the structural analysis using annular bright-field (ABF) high-resolution STEM near the BFO surface in Fig. 4e–g, where we can clearly see that Fe atoms moved upward (downward) once in contact with acidic (water or alkali) aqueous solutions, respectively. This dramatic atomic displacement indicates the direction of surface polarity reverses, which can be defined as^45^

2 $$P = \frac{e}{\Omega }\mathop {\sum}\nolimits_{m = 1}^N {Z_m^ \ast \Delta _{Z_m}}$$

where Ω is the volume of unit cell, *N* is the number of atoms in the unit cell, $Z_m^ \ast$ is the Born effective charge^46^ (calculated $\Delta Z_m^ \ast$ for Bi: 4.85, Fe: 4.10 and O: −2.61 along [110]). Here, Δ*Z*_*m*_ is the displacement of the atom away from its central symmetric position. Based on the calculations, we can obtain the polarization of surface layers for the above-mentioned four slab model (Supplementary Fig. 7), implying that ionic adsorption and interaction on the BFO surface provide a driving force to induce upward/downward bulk polarization switching.

In order to further phenomenologically understand the bulk ferroelectric switching, we performed phase-field simulations to demonstrate the polarization reversal based on the chemical adsorption-induced change of surface charge. For BFO films with downward polarization (positively charged surface), the surface would be negatively charged by OH^−^ adsorption, which is evidenced by our EFM measurement shown in Supplementary Fig. 6c, d, which results in an enhancement of overall electrostatic energy (see Methods). Our phase-field simulation demonstrates that the accumulation of negative (positive) charge on the BFO surface can lead to polarization reversal from downward (upward) to upward (downward) state, as shown in Fig. 4h, i. It further reveals that the polarization of a 30 nm thick BFO film can be reversed by an accumulation of surface charges with a density of ~ 10^12^ electrons/cm^2^. However, for BFO films with a thickness ≥ 90 nm (with high energy barrier for polarization switching), the polarization cannot be switched under the same surface charge density. This phenomenological insight is also supported by the thickness-dependent polarization switching as shown in Supplementary Fig. 8. In tradition, electrostatic energy can be stored in a body by charging it. The ionic introduction of H^+^/OH^−^ on the surface can induce the increase of the electrostatic energy and a decrease of the chemical energy, so that a controllable/dynamic charge transfer in accompany with an efficient energy conversion across the interface may be achieved. The present bulk polarization switching behaviors over the whole film within the volume are also directly evidenced by cross-sectional STEM imaging as shown in the Supplementary Fig. 9.

### Erasable type-printing of bulk polarization

**Fig. 5:**
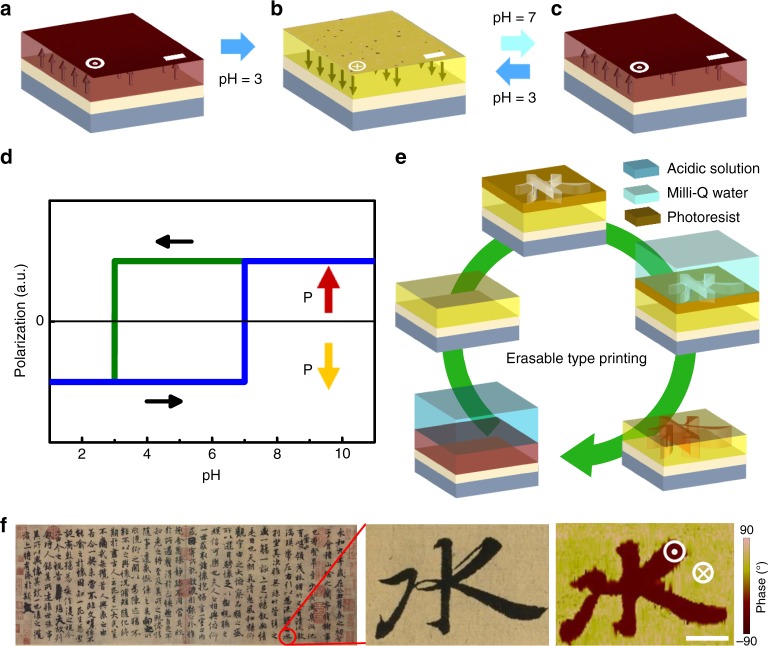
Erasable type-printing of bulk ferroelectric polarization. PFM images of an initially upward-polarized BFO grown on a STO substrate with a LSMO layer (**a**), the polarization reversed to downward after being exposed to acidic solution (pH = 3) (**b**) and cyclically switchable after being exposed to Milli-Q water and acidic solution (**c**). Scale bar, 2 μm (**a**–**c**). **d** Shows out-of-plane projections of the polarization direction vs. pH value of aqueous solution for initial upward-polarized BFO. **e** A schematic of printing and erasing the ferroelectric polarization by exposing the BFO to aqueous solution with different pH value. **f** PFM images of a Chinese characters 水 (water) from a calligraphy artwork^47^, designed by the printing process in **e**. Scale bar, 10 μm (**f**)

With a full understanding of the ionic interaction process, a deterministic control of the interplay between chemical and electrostatic energies could be an effective pathway towards the determination of interfacial structures and an in-situ reversible polarization switching of BFO in aqueous solution. Based on previous results, the interfacial chemical potential was mainly tuned by H^+^ or OH^−^ concentration near the BFO surface. Therefore, we discover that a BFO thin film with initial upward polarization can be switched to downward polarization once in contact with acidic aqueous solution (pH = 3 in whole solution), and then the downward polarization can be further switched back to its original upward state when this sample was in contact with Milli-Q water again (pH = 7 in whole solution), as shown in the PFM results in in Fig. 5a–c. Such a reversible bulk polarization switching can be achieved back and forth with the acidic aqueous solution and water treatments in sequence (Supplementary Fig. 10). This reversible ionic interaction switching process of polarization in BFO is summarized in Fig. 5d, demonstrating its potential to type print the polarization as desired nano-patterns in large-scale. As illustrated in Fig. 5e, the above reversible polarization switching combined with the micro/nano-lithography technique can help achieve erasable large-scale type-printing of ferroelectric polarization (details can be shown in Methods). Here, we use the greatest work of art in Chinese calligraphy “Preface to Orchid Pavilion” (created by Xizhi Wang and copied by Chengsu Feng in Tang Dynasty about 1400 years ago) as a templet in Fig. 5f, which can be certainly extended into multiple art formats^47^. One of the Chinese characters “水”, translated into “water”, has been printed out as reversed polarization using Milli-Q water and then acquired using PFM as shown in Fig. 5f, which demonstrates that ferroelectric polarization can be large-scale type printed into microscopic patterns using green energy. These results can not only give insights that physical and chemical energy inter-conversion may occur when surface chemistry meets condensed matter physics, but also pave the way to implant ferroelectric polarizations into applications such as prototype lab-on a chip microfluidic devices, bio-sensing, and nanoscale catalyzing etc. with low-cost manufacturing.

### Conclusions

We demonstrate an aqueous-solution-induced reversible switching of bulk polarization by controlling chemical bonds at the BFO surface. The large-area erasable type-printing of polarization is driven by the strong ionic interaction and consequent atomic displacement of Fe with respect to perovskite oxygen octahedral, which provides a brand-new strategy to achieve ferroelectric-based data storage and sensing devices. The inter-conversion of chemical and electrostatic energies across the ferroelectric/water interface offers controllable interfacial structures and dynamic charge transfer process via polarization switching, which may also build up a platform for high-efficient catalysis (overcoming the limitations imposed by the Sabatier principle^15^, where the surface chemical properties are not fixed but may be dynamically controlled by switching the polarization), energy storage, etc.

## Methods

### Epitaxial growth of BFO thin films

Epitaxial BFO thin films were grown using a pulsed laser deposition on atomically smooth (001) STO substrates with LSMO conductive buffers. Both LSMO and BFO films are deposited in an oxygen atmosphere of 20 Pa at a constant temperature of 700 °C. The growth rate is 1 nm/min and the cooling rate is 5 °C/min under an oxygen atmosphere were used. A laser energy density of 1.2 J/cm^2^ and a repetition rate of 5 Hz were used during the deposition. The thickness of the films was controlled at ~ 30 nm.

### Polarization switching and PFM measurements

The upward (downward) polarization switched to downward (upward) by scanning the surface with a tip under +5 V (−5 V) bias exceeding the coercive voltage. The polarization direction can be characterized by PFM, which is based on the detection of the bias-induced piezoelectric surface deformation. The PFM measurements were carried out on commercial scanning probe microscope (SPM) systems (nanoscope-V multimode AFM) equipped with external lock-in amplifiers (SRS 830, Stanford Research Instruments) under ambient conditions. The ferroelectric domain structures were imaged by a commercially available Pt-coated Si tips under an AC voltage which amplitude and frequency were controlled as 1 V_pp_ and 22 kHz. In this work, all the PFM phase images are out-of-plan phase images.

### Liquid PFM and high-resolution AFM measurements

Similar with the case at ambient conditions, liquid PFM measurements were performed by applying an AC voltage (1 V_pp_, 36 kHz) to a Pt-coated Si tip in contact with the BFO surface. A commercial liquid tip holder (Veeco) was used to bias the tip directly during contact with BFO surface in water. The PFM phase signal, which contains information about the orientation of the polarization of the sample beneath the tip, was captured in Milli-Q water as shown in Supplementary Fig. 1. The high-resolution AFM imaging were performed in tapping mode with oxide-sharpened Si_3_N_4_ cantilevers silicon cantilevers (spring constants between 36 and 75 N/m). The images were recorded in air, at room temperature and a scan rate of 1 Hz per line.

### XPS measurements

XPS measurements were carried out at room temperature in an ultra high vacuum system with the surface analysis system (ESCALAB 250Xi/ThermoFisher). A monochromatic Al Kα X-ray source (1486.7 eV), operating at 15 kV (90 W) was used for the measurement. The photoelectrons were analyzed at a take-off angle of 90°. High-resolution spectra were collected with analyzer pass energy of 20 eV. All spectra were calibrated according to the deviation between the C 1*s* peak and the standard binding energy of C 1*s* at 284.8 eV.

### First-principles calculations

First-principles calculations within the framework of density functional theory (DFT) are performed with VASP package^48^. We use the generalized gradient approximation (GGA) of Perdew, Burke, and Ernzerhof (PBE) for the exchange-correlation functional^49^. Pseudopotential of projector augmented wave (PAW) are employed^50^. The energy cutoff for plane waves is 500 eV. The centered 3 × 3 × 1 k points are used to sample the Brillouin zone. The tolerance for total energy convergence is 10^−4^ eV. Structure relaxation is converged until the forces on all atoms are less than 0.04 eV/Å. The lattice constant of bulk R3c BiFeO_3_ is 5.71 Å, in good agreement with the corresponding experimental one (5.63 Å). The energy gap of bulk R3c BiFeO_3_ is 2.1 eV, also in good agreement with the corresponding experimental one (1.9 eV)^51^. The Coulomb interaction *U*_eff_ = 4 eV is applied on Fe atoms. The Fe atoms exhibit antiferromagnetism both at intra-layers and inter-layers.

### High-resolution STEM measurement

The crystal structures of the BFO were performed using a JEOL 2100F (JEOL, Tokyo, Japan) transmission electron microscope operated at 200 keV and equipped with CEOS (CEOS, Heidelberg, Germany) probe aberration corrector, double spherical aberration (Cs) correctors. The spatial resolution of the microscope is 90 pm at the incident semi-angle of 20 mrad. The image simulation was carried out based on a fast-Fourier-transform multi-slice approach for the STEM configuration.

### EFM measurement

The EFM is implemented on commercial SPM systems (Nanoscope-V Multimode AFM) with tapping mode. To detect the electrostatic forces, a voltage is applied to AFM tips (−1 V) coated with the Pt metal which are scanned across the surface with a constant tip-sample separation (80 nm). The surface charge can be detected with phase differences induced by electrostatic forces on the oscillating tip during scanning.

### Phase-field simulations

Ferroelectric polarization switching in BFO thin films was simulated using the phase-field model by solving the time-dependent Ginzburg-Landau (TDGL) equation for the temporal evolution of the polarization vector field,

3 $$\frac{{\partial {\mathbf{P}}_{\boldsymbol{i}}\left( {{\mathbf{r}},t} \right)}}{{\partial t}} = - L\frac{{\delta F}}{{\delta {\mathbf{P}}_{\boldsymbol{i}}\left( {{\boldsymbol{r}},t} \right)}},i = 1,2,3$$

where **P**_***i***_(**r**, *t*) is the polarization, *L* is a kinetic coefficient that is related to the domain wall mobility, and *F* is the total free energy that includes contributions from the Landau energy, elastic energy, electric energy, and gradient energy.

4 $$F = {\int\!\!\!\!\!\int\!\!\!\!\!\int} {\left( {f_{{\mathrm{Landau}}} + f_{{\mathrm{elastic}}} + f_{{\mathrm{electric}}} + f_{{\mathrm{grad}}}} \right){\mathrm{d}}V}$$

Detailed descriptions of the total free energy *F* were presented in some depth in our previous publication^52^. The Landau, elastic and electrostrictive coefficients used are described in the reference^53^. The equation was solved by a semi-implicit Fourier spectral method using a discreet grid of 256 Δ*x* × 1 Δ*x* × 128 Δ*x* (Δ*x* is the number of grid points and equals to 1 nm in this work) with the periodic boundary conditions along x and y directions in the film plane. We assume that the electric potential at the bottom of thin film is zero because of contacting with the conducting substrate which is set as the ground, while at the top of thin film the electric potential is assumed to be continuous.^54–56^.

$$\Phi \left| {\,_{z = 0}} \right. = 0,\,\Phi \left| {\,_{z = h - 0}} \right. - \Phi \left| {\,_{z = h + 0}} \right. = 0$$

5 $$\left( {{\mathbf{P}}_z - \varepsilon _0\varepsilon _b\frac{{\partial \varphi }}{{\partial z}}} \right)\left| {\,_{z = h - 0}} \right. + \varepsilon _0\varepsilon _b\frac{{\partial \varphi }}{{\partial z}}\left| {\,_{z = h - 0}} \right. + \sigma _{{\mathrm{surface}}} = 0$$

In addition, we only consider surface charge density *σ*_surface_ at the top of thin film. The electrostatic potential *φ*(*r*) is described by:

6 $$- \nabla ^2\varphi = \frac{{\sigma _{s{\mathrm{urface}}} - \nabla \cdot {\mathbf{P}}}}{{\varepsilon _0\varepsilon _r}}$$

where *ε*_0_ is the permittivity of free space, and *ε*_*r*_ is the permittivity of BFO.

### Erasable large-scale type-printing of ferroelectric polarization

Polarization printing process were demonstrated in Fig. 5e, the sequence involves switching the initial upward polarization to downward by acidic-solution firstly, then creating a patterned coating on the BFO surface by photoengraving, reversing the downward polarization of exposed polar surface to upward direction with water treatment and removing the photoresist. Taking advantage of photolithography methods, we can achieve large-scale ferroelectric polarization printing rapidly and effectively.

## Electronic supplementary material

Supplementary Information

## Data Availability

The authors declare that the data supporting the findings of this study are available within the paper and its Supplementary Information Files.
