# Supplementary material for: Water printing of ferroelectric polarization
Source: Nat Commun. 2018 Sep 18;9:3809. doi: 10.1038/s41467-018-06369-w (PMC6143547; doi:10.1038/s41467-018-06369-w)
Supplement: Supplementary file 1 — Supplementary Information [file 41467_2018_6369_MOESM1_ESM.pdf]

Water Printing of Ferroelectric Polarization

Tian et al.

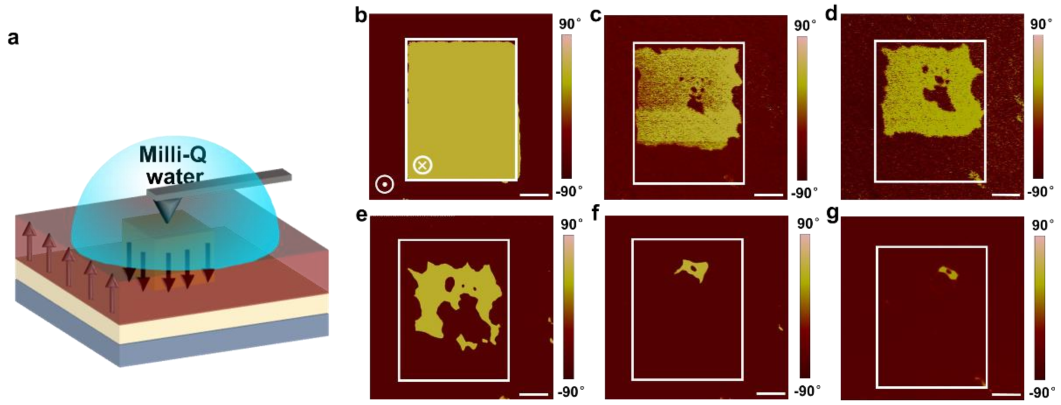

Supplementary Figure 1 | Evolution of polarization switching in water. a, A schematic of liquid piezoresponse force microscopy (PFM) measurement in Milli-Q water. Here, yellow box at middle region is the written ferroelectric domain by tip bias, and the arrows describe the out-of-plane component of the polarization. **b**, PFM phase images of the domain pattern electrically written by the scanning probe applied with +5 V bias. **c-g**, PFM phase images of the same area with (**b**) after exposing the film in water for 30 min (**c**), 50 min (**d**), 70 min (**e**), 90 min (**f**), 120 min (**g**), respectively. Scale bar, 1 μm (**b-g**).

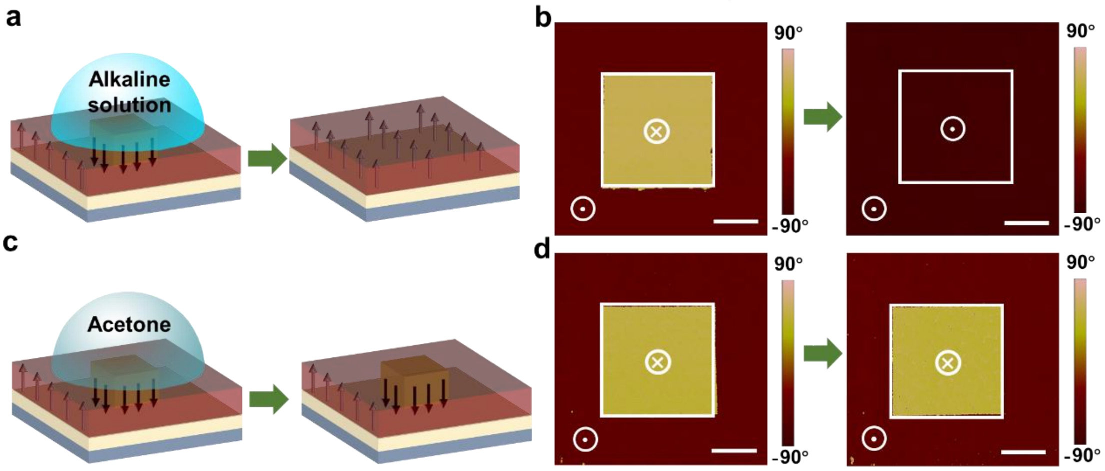

Supplementary Figure 2 | Alkaline-solution and acetone treatments. a,c, Schematics of $BiFeO_3$ (BFO) grown on a $SrTiO_3$ substrate with a (La,Sr)MnO_3 layer, polarization switching from downward to upward after alkaline-solution treatment (**a**) and no polarization switching after acetone-solution treatment (**c**). **b**,**d**, Corresponding PFM phase images of the $BiFeO_3$ (BFO) films before and after alkaline-solution (**b**) and acetone (**d**) treatment. Scale bar, 2 μm (**b**, **d**).

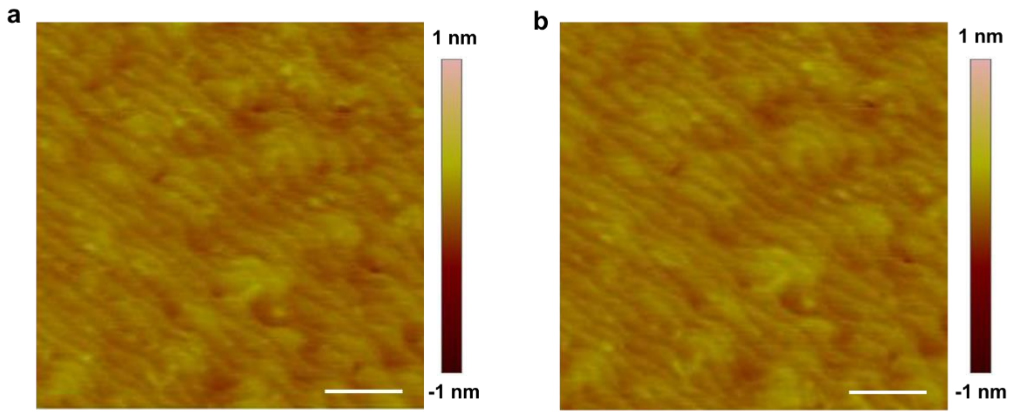

Supplementary Figure 3 | No change of BFO surface morphology after acidic-solution treatments. The topography of BFO thin film before (**a**) and after (**b**) acidic-solution-induced polarization reversal. Scale bar, 500 nm (**a**, **b**).

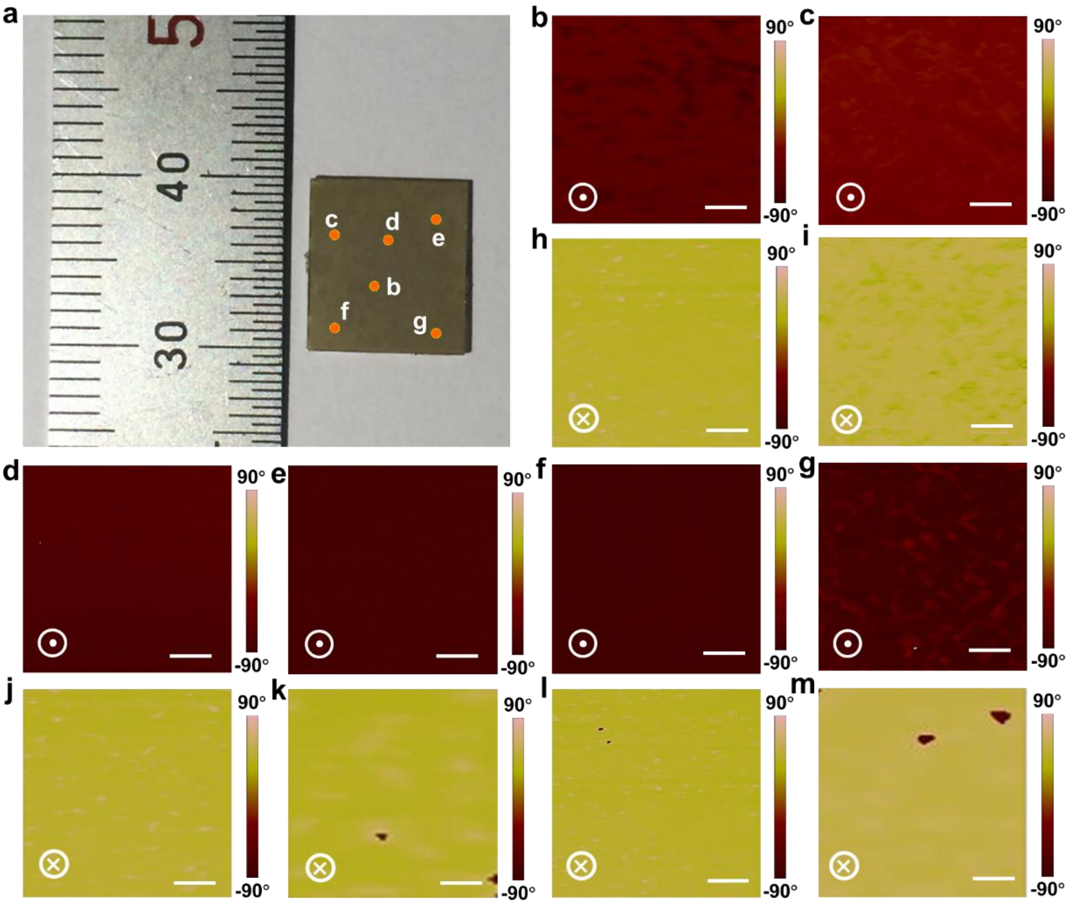

Supplementary Figure 4 | Polarization switching of the whole BFO thin film. The switched sample size can be shown in (**a**) and the polarization directions (**b-g**) examined by PFM (every dots correspond to the figures). (**h-m**) The switched PFM phase images at same location corresponding to (**b-g)** after acidic-solution treatment (pH=3). Scale bar, 1 μm (**b-m**).

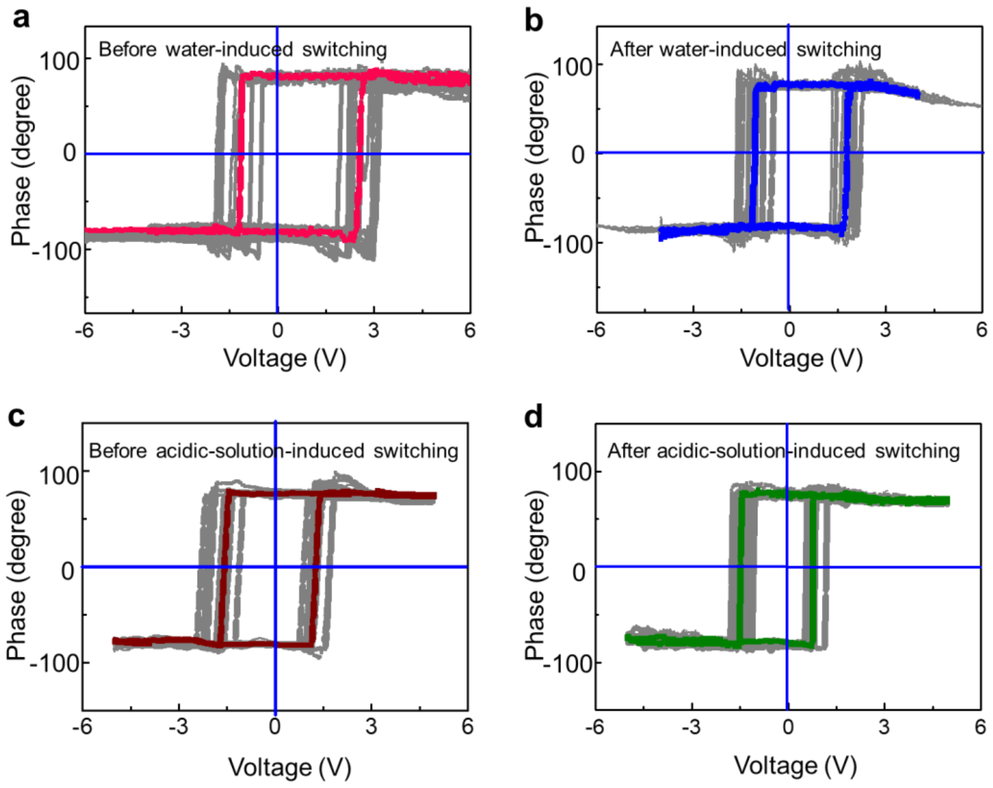

Supplementary Figure 5 | Evolutions of the BFO coercive field after aqueous-solution-induced polarization switching. Piezoresponse hysteresis loops of BFO films with initial upward polarization states (**a**), after Milli-Q water-induced switching (**b**). Piezoresponse hysteresis loops of BFO films with initial downward polarization states (**c**), after acidic-solution-induced switching (**d**). Here, gray curve lines show the raw data from multiple measurements, color curve lines display the average values.

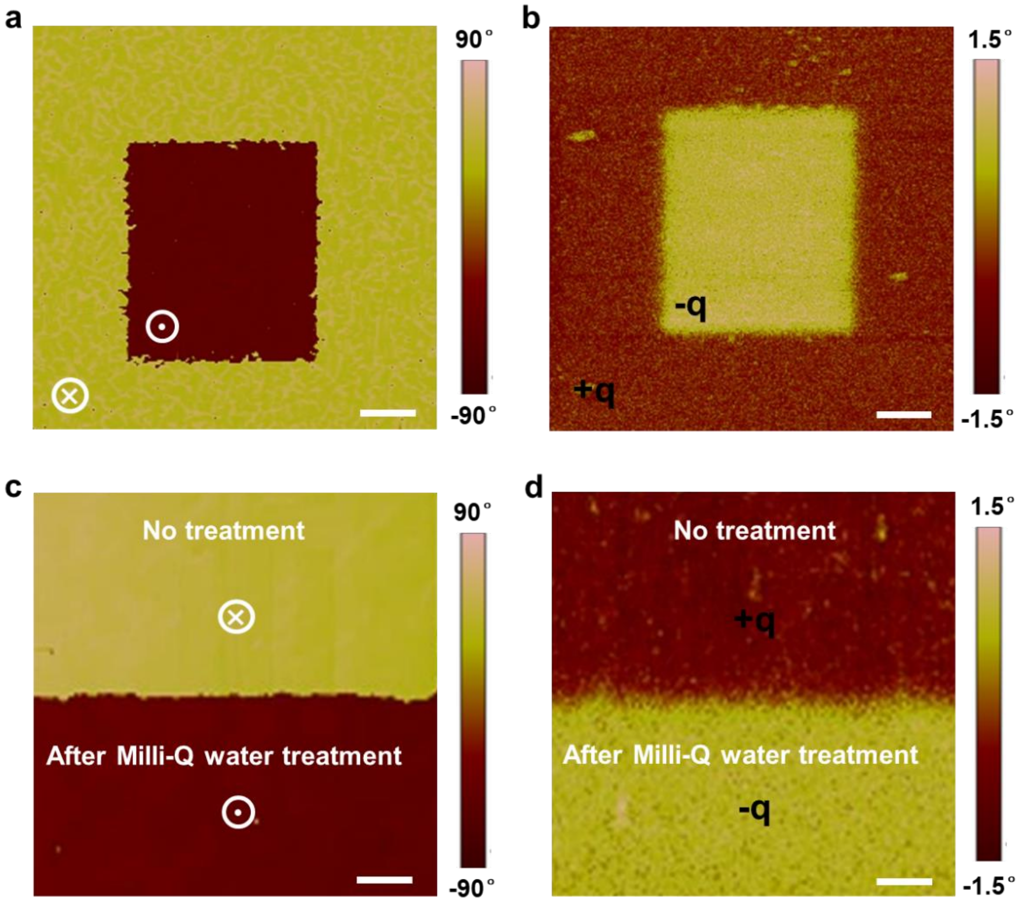

Supplementary Figure 6 | PFM (left) and electrostatic force microscopy (EFM) (right) images of BFO thin film. **a**, PFM phase of whole BFO thin film with downward polarization state, and the middle box was written by tip bias for comparison. **b**, Corresponding EFM image shows that the downward-polarized BFO surface are positively-charged (measured with tip bias -1 V). **c**, PFM phase of BFO thin film with polarization switched to upward state after being exposed to Milli-Q water, and the upper portion of the image is the area of the film that did not exposed to water. **d**, Corresponding EFM image shows that the BFO surface charge changed from positive to negative after water-induced polarization switching (measured with tip bias -1 V). Scale bar, 2 μm (**a**-**d**).

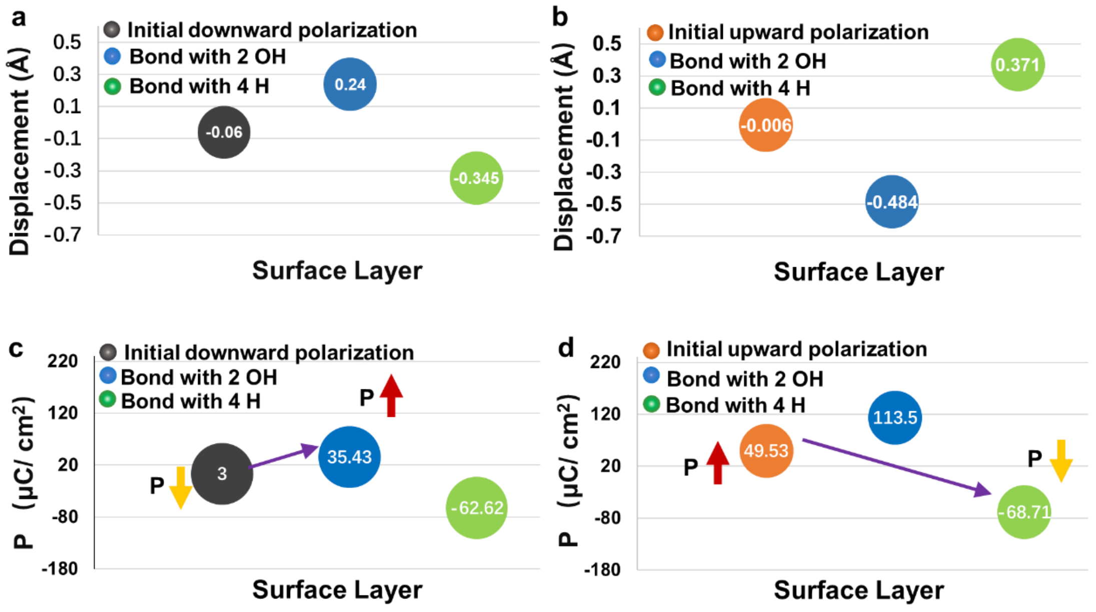

Supplementary Figure 7 | Atom displacements and polarizations of first-principle calculations. **a**, The local surface displacements *D* (between intra-layer anions and cations) with initial upward-polarized BFO, bonding with four H, and two OH. **b**, The local surface displacements *D* with initial downward-polarized BFO, bonding with four H, and two OH. **c**, **d**, Corresponding surface polarization *P* defined by equation (2) of the main text.

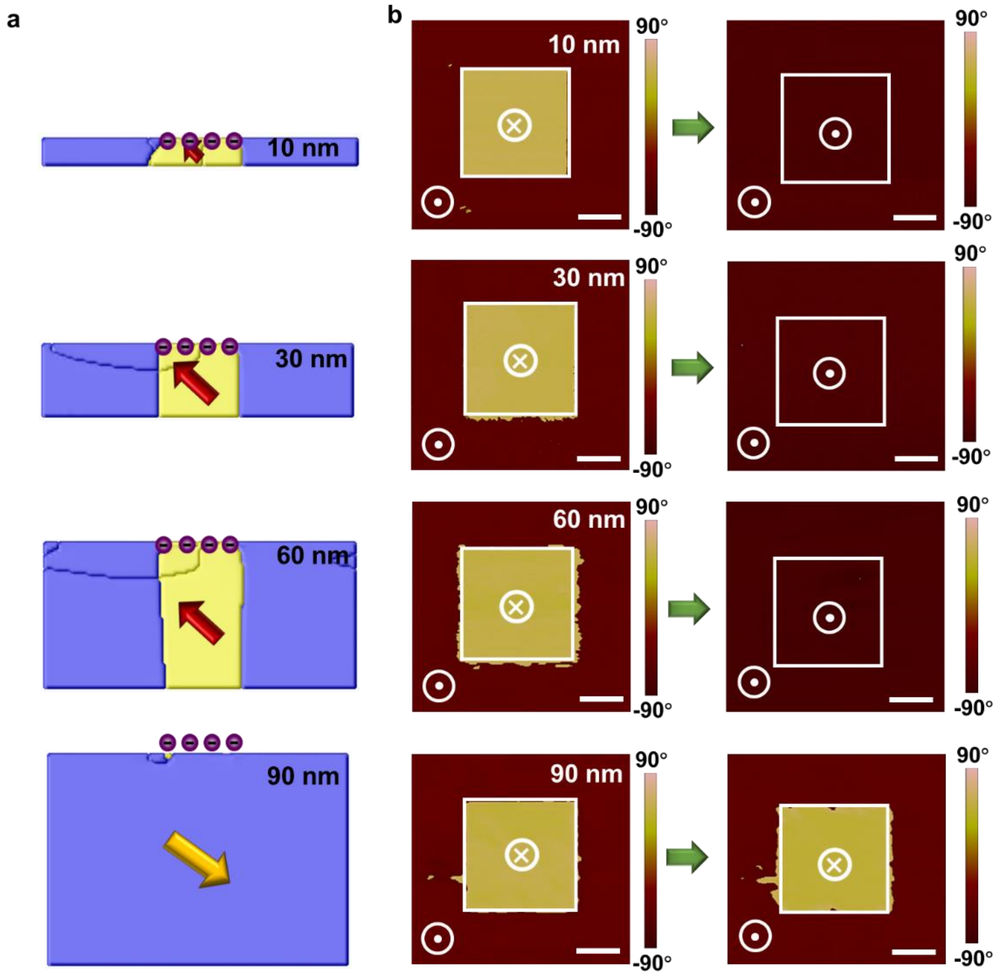

Supplementary Figure 8 | Thickness dependence of water-induced polarization switching. (**a**) Initial downward-polarized BFO film after adding an electric charge with 10^{12} cm^{-2} at the surface with different thickness, including 10 nm, 30 nm, 60 nm and 90 nm. (**b**) Corresponding PFM phase images of initial upward-polarized BFO films with an inside downward-polarized domain (5×5 μm^2) after water treatment. After exposing in water, polarization switched from downward to upward for the thickness of BFO film below 90 nm, as demonstrated by the PFM images. Scale bar, 2 μm (**b**).

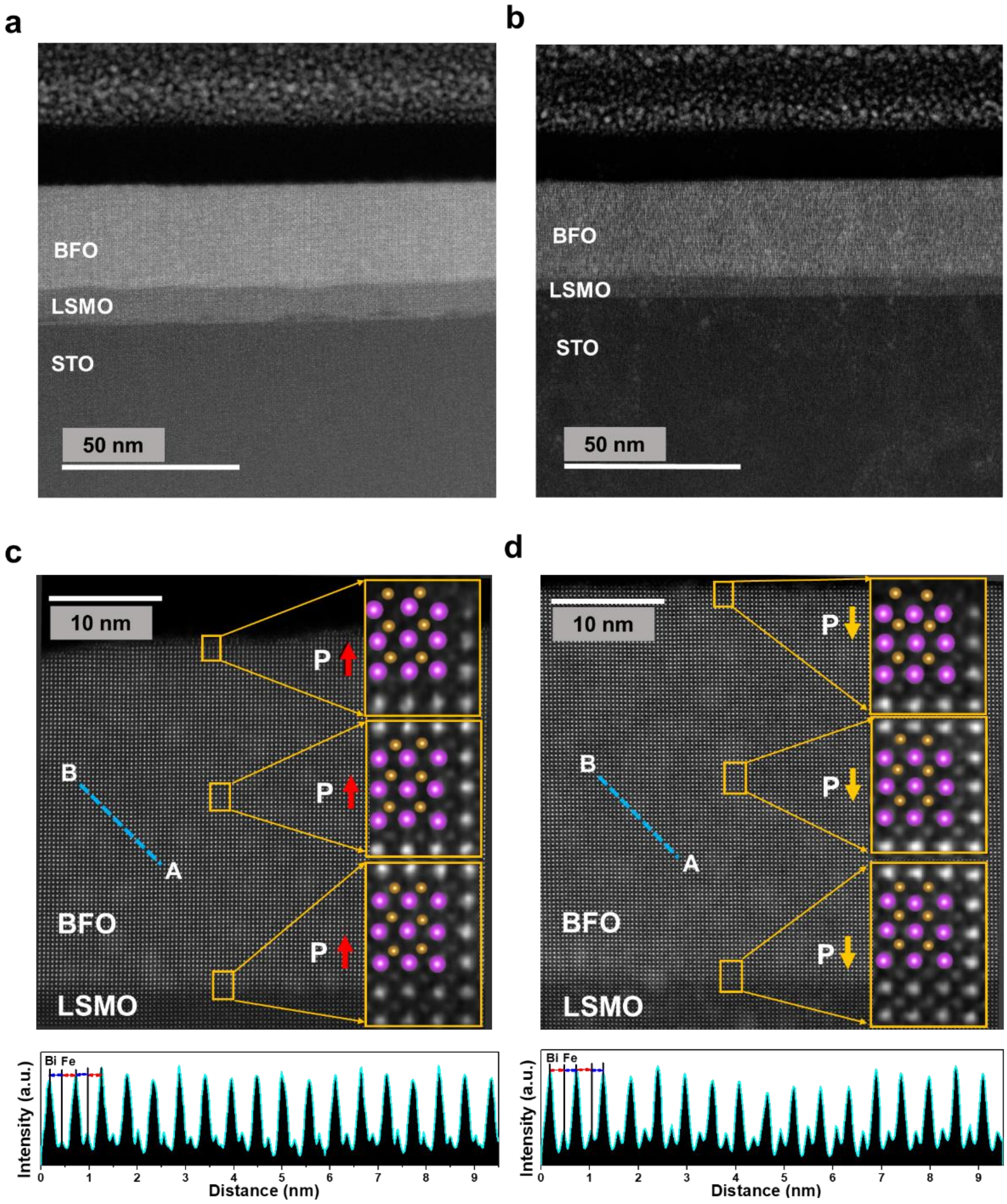

Supplementary Figure 9 | Bulk polarization switching induced by Milli-Q water and acidic solution. a, **b**, Low mag scanning transmission electron microscopy (STEM) images of BFO thin film after polarization switching induced by Milli-Q water (**a**) and acidic aqueous solution (**b**), indicating there is no unswitched domains buried underneath the surface. **c**, **d**, STEM images of the whole BFO thin films after polarization switching induced by Milli-Q water (**c**) and acidic aqueous solution (**d**). The red and yellow arrows represent the out-of-plane component of the polarization. Intensity line profiles generated from the bulk of BFO thin film (dashed blue lines (from A to B)), showing opposite shifts of the Fe with respect to the Bi.

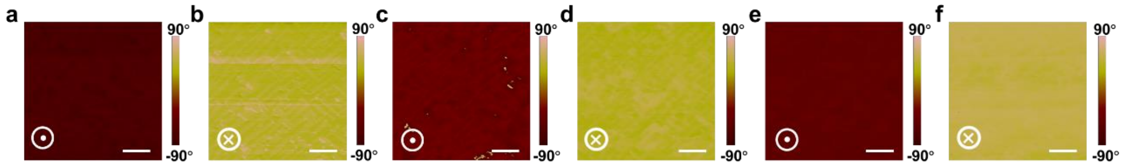

Supplementary Figure 10 | Reversibly bulk polarization switching by aqueous-solution treatments. a, PFM phase images of initial upward-polarized BFO film. **b-f**, The controllable polarization switching cycle can be achieved by designing chemical bonds across polar surface and aqueous solution: the polarization switched to downward after immersing it in acidic solution with pH=3 (**b**, **d**, **f**) and reversed back to upward by exposing the sample to Milli-Q water (**c, e**). Scale bar, 1 μm (**a-f**).
